# Rock, paper, scissors: Resolving the conflicting results of randomized trials involving corticosteroid, platelet rich plasma (PRP) and placebo injections

**DOI:** 10.1016/j.jsampl.2024.100081

**Published:** 2025-01-02

**Authors:** John W. Orchard

**Affiliations:** School of Public Health, University of Sydney, Sydney, Australia

**Keywords:** Knee osteoarthritis, Rotator cuff tendinopathy, Tennis elbow, Plantar fasciitis, Injections

## Abstract

**Objective:**

To explore whether there is evidence of a rock-paper-scissors phenomenon in injections for various musculoskeletal conditions whereby platelet rich plasma (PRP) injections outperform corticosteroid injections (CSI) in randomized trials, corticosteroid outperforms placebo whereas PRP does not outperform placebo.

**Methods:**

Narrative review searching for examples of musculoskeletal conditions which have high level evidence of this proposed rock-paper-scissors phenomenon.

**Results:**

High quality studies or reviews could be found for lateral epicondylitis (tennis elbow), knee osteoarthritis, rotator cuff tendinopathy and plantar fasciitis suggestive of a rock-paper-scissors phenomenon. This was consistently in the direction of PRP injections having superior results to corticosteroid injections, corticosteroid injections having superior results to placebo, but then evidence that PRP injections were not superior to placebo. The most consistent result of the triumvirate was that PRP injections consistently outperformed corticosteroid injections in the medium-term (4 to 12 months).

**Discussion:**

There appears to be somewhat of a rock-paper-scissors phenomenon for four common musculoskeletal conditions, with the significant limitation that this was a finding of a narrative review, limited by a non-systematic search protocol. The underlying explanation is that PRP is superior to corticosteroid in the medium-term (but not the short-term), corticosteroid is superior to placebo in the short-term (only) with PRP not being superior to placebo in either short- or medium-term in well-blinded trials. The most likely explanation for this phenomenon is that corticosteroid injections are helpful for musculoskeletal conditions in the short term (≤8 weeks) but are actually harmful in the medium-term (3 months and beyond). Systematic reviews which further explore this phenomenon are justified to help provide practical evidence-based advice on when to, if ever, use PRP and corticosteroid injections for musculoskeletal conditions.

## Graphical abstract

Sample progress (y-axis showing vertical improvement) between PRP (red), corticosteroid (green) and placebo (blue) over x-axis timeframe of months. Corticosteroid injections (CSI) give the best results in the first 2 months followed by the worst results in the medium term (6–12 months).

**Figure:**
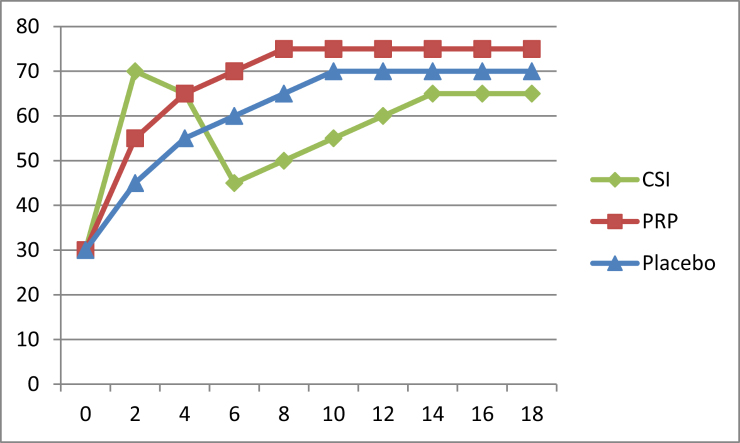

## 1 Introduction

Injections are commonly used in musculoskeletal medicine to relieve pain symptoms from both joint and soft tissue pathology. The most commonly-used injection is corticosteroid which has a history dating back more than 60 years, with controversy evident from the earliest days [1]. The number of injection types and postulated indications has increased rapidly over the last few decades, although knee osteoarthritis is one of the most common indications. The second most-investigated injection type in the modern era is Platelet Rich Plasma (PRP), with PRP injections the most common representative of “biologic” or “autologous” injection category. For the latter years of the 20th Century, injections were encouraged by apprenticeship and mentoring, in the absence of much high-quality evidence. Fortunately this new century has finally seen a proliferation of Randomized Control Trials (RCTs) to assess the efficacy of many of the injection types for various indications.

The frustration of the current era is different: we *do* have many high-quality RCT-based trials regarding various injections. The modern problem is that the results of RCTs are not easily collated into systematic reviews and meta-analyses which seem consistent with each other. If the indication for an injection in the 20th Century required a faith-based approach on behalf of the doctor because of the lack of RCTs, in the 21st Century a faith-based approach is still required because of conflicting evidence of various systematic reviews. Some of the data in this paper was presented at a conference panel in Melbourne in October 2024: an audience question that was very pertinent was “when there is one panel member suggesting to use corticosteroids, one suggesting to use a biologic like PRP and one panel member suggesting not to inject, how are we to interpret this advice?” The answer is that there seems to be no strong evidence that makes one of these options clearly correct.

Knee osteoarthritis systematic reviews appear as a *non-sequitur*. Many of the major guidelines for knee osteoarthritis, for example [2], recommend corticosteroid as a treatment for knee osteoarthritis based on high-quality evidence showing corticosteroid injections having superior outcomes to placebo injections [3,4]. The same guidelines recommend against PRP as a treatment for knee osteoarthritis based on a perceived lack of proven benefit against placebo (saline) injection, as evidenced by some high-quality recent placebo-controlled RCTs [5,6]. However, there are meta-analyses which combine trials where PRP has been directly compared to corticosteroid in randomized trials, and these trials decisively conclude that PRP demonstrates superior results to corticosteroid [7]. It seems impossible that corticosteroid is superior to placebo, that placebo is non-inferior to PRP but also that PRP is superior to corticosteroid; however this seemingly discordant set of results is suggested by high quality evidence of knee osteoarthritis.

Furthermore, knee osteoarthritis is not the only condition where this three-way contest seems to result in one win and one loss for each of the three options of corticosteroid, PRP and placebo. This is reminiscent of the game of Rock, Paper, Scissors where rock beats (blunts) scissors, scissors beats (cuts) paper but paper beats (covers the) rock.

The objective of this discussion paper is therefore twofold: firstly, to attempt to find multiple musculoskeletal conditions where a rock-paper-scissors phenomenon might be present. Secondly, to the extent that this phenomenon gets demonstrated, to try to explain how it could possibly come about when it seems to make no sense. An understanding of the origin of apparent discordance between different systematic reviews may help the clinician (and patient) decide when to consider injection therapy with corticosteroid or PRP and when to reject it.

## 2 Methods

The primary analytical method is a narrative review of high-quality evidence (either systematic review, meta-analysis or single excellent quality randomized control trial). For multiple musculoskeletal diagnoses, I aimed to find high-quality evidence, indexed on PubMed which contained RCT-based comparison of all of (1) PRP vs corticosteroid trials (2) corticosteroid vs placebo trials (3) PRP vs placebo trials.

The final inclusion criteria was that I was only looking for examples of what I have called a rock-paper-scissors phenomenon, whereby PRP, corticosteroid and placebo won one arm and lost one arm of the three head-to-head comparisons (of which they participated in two each).

The primary objective was to look for rock-paper-scissors outcomes in musculoskeletal conditions. The hypothesis was that rock-paper-scissors sets would exist and that they would always exist in the same direction (PRP defeating corticosteroid, corticosteroid defeating placebo injection and placebo injection defeating or neutralizing PRP).

The second objective of this narrative paper was to describe possible mechanisms for any rock-paper-scissors phenomenon that was observed from the outcome of the review.

## 3 Results

### 3.1 Which conditions had evidence of a rock-paper-scissors phenomenon between PRP, corticosteroid and placebo?

Four conditions were found where there was as least moderate-quality evidence of a rock-paper-scissors phenomenon as described (in Table 1) although the phenomenon was not compelling for any of the four.

**Table 1:** Narrative head-to-head comparisons between three injection options for four conditions.

Condition	Evidence showing PRP being superior to corticosteroid	Evidence showing corticosteroid being superior to placebo	Evidence showing placebo to be non-inferior to (defeating) PRP	Conclusion (and direction), with disclaimer
Tennis elbow	Li et al. [8], 7 RCTs; PRP better medium-long term results and overall success	Corticosteroid generally superior results in short term (only) [9].	Simental-Mendia et al. [10] 5 RCTs, no strong benefit seen for PRP injections	PRP ​> ​corticosteroid; corticosteroid ​> ​placebo in short term (but worse in medium term); placebo ​= ​PRP
Knee OA	McLarnon et al. [7], 8 RCTs, PRP had superior results to corticosteroid from 6 to 12 months	Najm et al. [4], meta-analysis of 15 RCTs, similar to Juni et al. [3]	2 recent high-quality RCTs show placebo ​= ​PRP [5,6] although trend otherwise to PRP ​> ​placebo [11]	PRP ​> ​corticosteroid; corticosteroid ​> ​placebo in short term; Placebo ​= ​PRP in high quality trials
Plantar fasciitis	Three meta-analyses [[12], [13], [14]]; PRP had superior results to corticosteroid from 6 to 12 months	Ang et al. 10 RCTs [15] and Li et al. [16] 4 RCTs showing short-term superiority of corticosteroid	Single double-blinded RCT showing no effects of PRP over placebo [17].	PRP ​> ​corticosteroid; weak evidence corticosteroid ​> ​placebo; one RCT showing placebo ​= ​PRP
Rotator cuff tendinopathy	Pang et al. [18], 13 RCTs; PRP superior to corticosteroid in medium term although not in short-term	Mohamadi et al. [19] showing superior results for corticosteroid from 4 to 8 weeks	Single RCT [20] although perhaps weak evidence of benefit [21].	PRP ​> ​corticosteroid; corticosteroid ​> ​placebo; one RCT showing placebo ​= ​PRP

For all four conditions, the strongest evidence consistently suggested that PRP injections were more effective than corticosteroid injections. There was weaker evidence that corticosteroid was superior to placebo (e.g. for tennis elbow this was only in the short-term, and results were worse in the medium term). There was generally moderate evidence suggesting that PRP injections were no better than placebo. The value of injections in general varied across the 4 conditions. Injections in general (of both types) seemed to be relatively better than placebo for rotator cuff conditions (although still with some evidence of the rock-paper-scissors phenomenon); for plantar fasciitis there was less evidence, albeit some, of either injection type beating placebo.

### 3.2 How can it possibly be that PRP beats corticosteroid which beats placebo which negates PRP?

The rock-paper-scissors phenomenon is partially true, although as surmised it would almost be a *non-sequitur* if it was completely true. Although placebo did not ever “beat” PRP in any trials, a tie was considered good enough for a win in that an active agent should theoretically outperform a placebo injection. By comparison, corticosteroid *does* generally outperform placebo injection in the short-term, coming across in this sense as superior to PRP, although some medium-term studies, especially for tennis elbow, actually show placebo outperform cortisone [22]. The paradox is most clearly outlined when in direct head-to-head studies, PRP outperforms corticosteroid for all of the four conditions.

**Fig. 1:**
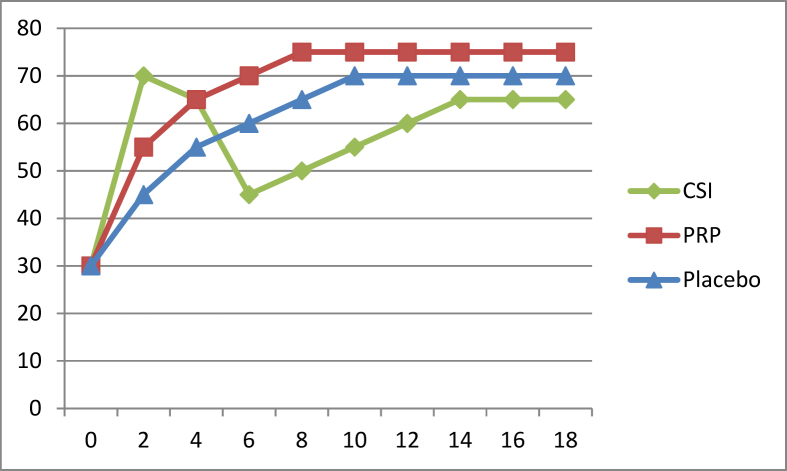
Sample progress (y-axis showing vertical improvement) between PRP (red), corticosteroid injection (CSI) (green) and placebo (blue) over x-axis timeframe of months.

Figure 1 shows a fictitious dataset but one which graphically reveals how this paradox probably occurs. The biphasic response of corticosteroid, which gives excellent results in the short-term but poor results (marginally worse than placebo) in the medium-term is the likely main driver of the paradox. PRP may have a slight but consistent superiority to placebo, but one which generally may fail to deliver either statistical significance or surpass minimum clinically-important difference. However, the slight advantage that PRP has over placebo in the short-term may be enough to stay within reach of corticosteroid, which it then overtakes in the longer-term.

**Fig. 2:**
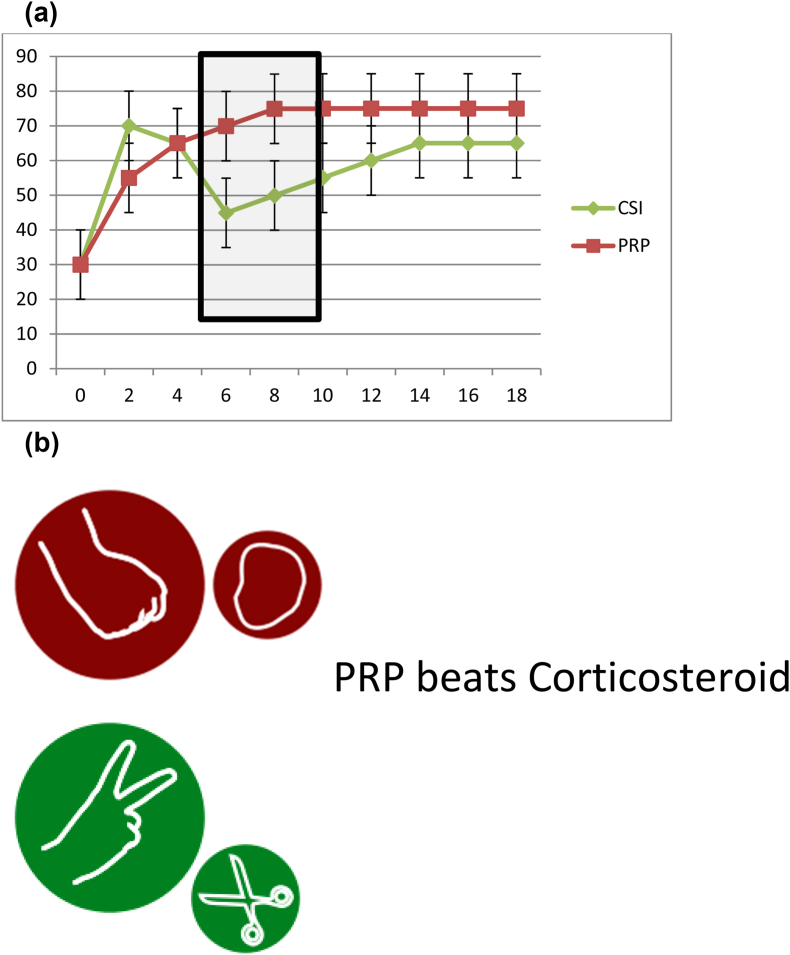
(a) Using the sample data from Fig. 1, example outcome of a PRP vs corticosteroid RCT over months. Fig. 2 (b) Sample interpretation: PRP significantly better outcome than corticosteroid at 6 months and 8 months, no significant differences at other times.

**Fig. 3:**
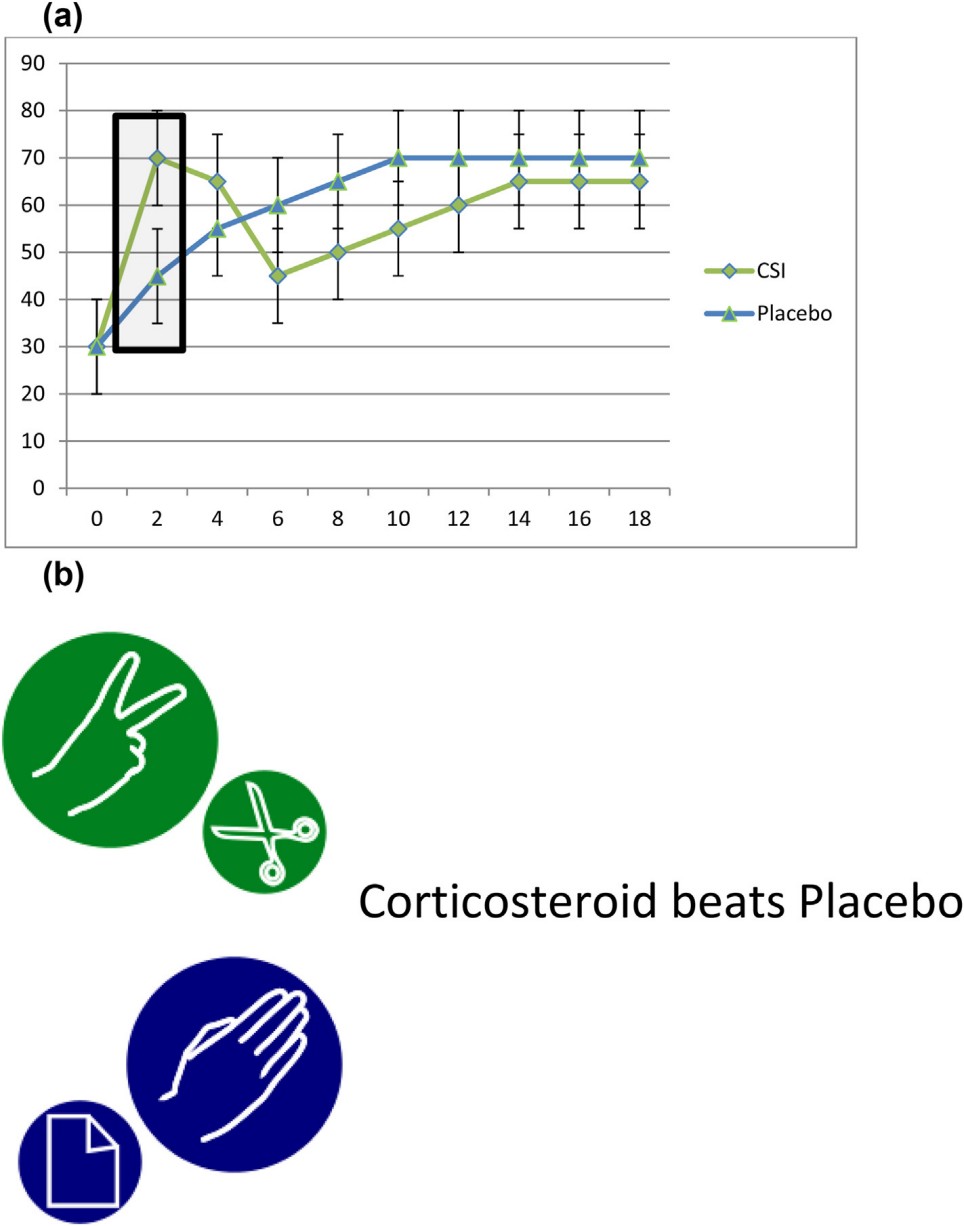
(a) Using the sample data from Fig. 1, example outcome of a corticosteroid vs placebo RCT. Fig. 3 (b) Sample interpretation: corticosteroid significantly better outcome than placebo at 2 months, no significant differences at other times.

**Fig. 4:**
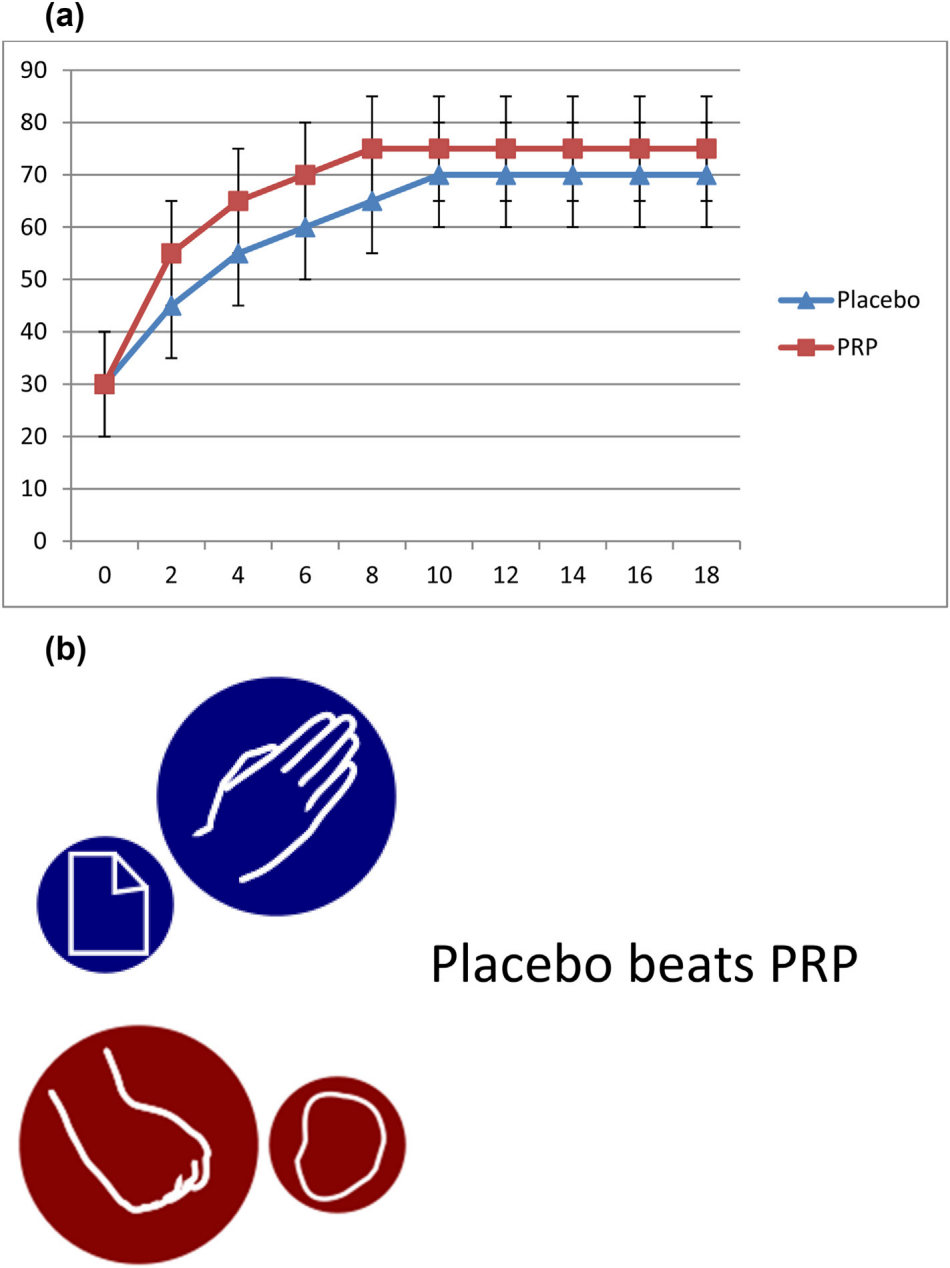
(a) Using the sample data from Fig. 1, example outcome of a PRP vs placebo RCT. Fig. 4 (b) Sample interpretation: No significant differences between PRP and placebo at any time, failing to establish inferiority of placebo.

Figure 2(a) and (b), 3 (a) and 3(b) and 4(a) and 4(b) break down Fig. 1 into three different head-to-head comparisons, again with fictitious error-bar data included, showing how the rock-paper-scissors outcome can appear from the dataset in Fig. 1.

## 4 Discussion

### 4.1 Strengths and weaknesses of this analysis

This paper is limited at being able to make strong conclusions about the effects of these injection types for the example conditions presented, in that a single narrative overview cannot reach the breadth of analysis that would be required to do justice to each condition. However, the strength of this paper is to take a holistic view of a major trend. Something strange appears to be going on from a distance whereby the results of the three head-to-head comparisons appear discordant. The rock-paper-scissors analogy appears to be partially valid. This review also only considered four of the most common musculoskeletal conditions, not necessarily representative of all degenerative musculoskeletal injuries.

### 4.2 The placebo effect of injections is very strong

Anyone inclined to view injections cynically may well draw the conclusion that if placebo is performing creditably against the two most-commonly used injection types that perhaps injections are of no benefit. Certainly a balanced view might be that they (injections) are over-rated. However, if much of the effect of injections is non-specific (AKA placebo) it still represents a potentially beneficial treatment which may in some cases compare favourably to other options. There is a strong surgical placebo effect as well [23], but with surgery being far more costly, risky and harmful to the environment.

Proponents of injections may argue that a saline injection, whilst considered control (placebo) in most RCTs, may well have some therapeutic effect. For example, exercise therapy, which is considered the mainstay of musculoskeletal treatment, may not outperform saline injection in direct head-to-head comparison either [24]. It remains debatable whether the benefits of saline injection (and indeed PRP) represent natural history that would have occurred without any injection or whether there is a partial therapeutic effect of injecting inert fluid.

PRP was a focus of this review because of the larger number of available trials than other injection options. It is plausible that hyaluronan, dextrose prolotherapy, stem cell, whole blood, cultured tenocytes and chondrocytes and bone marrow aspirate injections may all have similar relationships with corticosteroid and placebo injections to PRP. To date, no injection options for musculoskeletal conditions have been found to be “disease-modifying”. That is, if and when they work, the mechanism is reduction of pain rather than superior restoration of normal anatomy.

### 4.3 Any study involving corticosteroid must take into account late negative effects

Corticosteroid has become popular and a standard treatment for the majority of musculoskeletal conditions based on its short-term beneficial effects on pain [25]. It is becoming more clear that the early beneficial effect of corticosteroid is the earliest of a biphasic response whereby corticosteroid does less well than comparators in the medium-term. In order to justify a corticosteroid injection, the biphasic response for the condition being studied needs to be documented and taken into account, along with the patient needs in the very-short term versus medium to long term risks. There is evidence of emerging harm in the longer term for corticosteroid for knee and hip osteoarthritis in particular [[26], [27], [28], [29], [30], [31]].

Although we consider the double-blinded RCT to be the platinum standard of evidence, difficulties in blinding may pollute corticosteroid studies in particular. It is possible even in a well-conducted double-blinded corticosteroid vs placebo injection study that many subjects are aware of being in the intervention group because of the systemic side effects of corticosteroid in the first 48 ​h. In a study where the subjects have been told that there is one “control” (placebo) group, there may be a beneficial effect of feeling that one has successfully been randomised to the intervention group. This effect may be lesser in a study comparing corticosteroid to PRP where the subjects have been told that both groups represent a therapeutic intervention.

### 4.4 The greatest non-sequitur is society guidelines recommending in favour of corticosteroid injections but against PRP injections

From this narrow overview of the topic of injections, the strongest conclusion is that for the conditions studied, PRP injections perform better than corticosteroid injections. Societies cannot continue to ignore the head-to-head trials between PRP and corticosteroid. Table 2 shows that the only completely illogical stance is the one most societies take: to recommend against PRP but to also recommend corticosteroid [2,25,32]. Societies need to decide whether they believe PRP is an elaborate placebo (possibly the most likely reality, given the lack of large benefit in RCTs of PRP vs placebo). If indeed PRP is an elaborate placebo, it is OK to recommend against using it; but you must then also recommend against using corticosteroid, as corticosteroid fails to beat the elaborate placebo. With hindsight, a saline placebo which gives no side effects might be insufficient to rule out non-specific effects, and PRP – if considered a placebo - might be a better way to assess corticosteroid.

**Table 2:** Flawed logic of society guidelines given that PRP is superior to corticosteroid injections in almost every systematic review directly comparing these two options.

	Recommend PRP injection	Recommend against PRP injection
Recommend corticosteroid	Plausible stance (corticosteroid for short-term; PRP for longer-term)	Implausible stance (but taken by society guidelines)
Recommend against corticosteroid	Plausible stance	Plausible stance (neither better than placebo in the longer term)

If societies see some benefit in placebo injections due to these non-specific effects, which is also a reasonable stance, then they also must rate PRP injections, and potentially other types of injections, as options if they are recommending corticosteroids.

### 4.5 Registries are required to examine the long term (2 years +) effects of injections

A final consideration is that very few studies to date (including the vast majority of RCTs) examine outcomes beyond 2 years. It is important to determine whether PRP and corticosteroid are helpful, harmful or neutral in the longer-term, particularly for conditions that have a significant treatment failure rate at two years or beyond like hip and knee osteoarthritis. Registries of patient outcomes are likely to provide more knowledge than RCTs for the long-term effects of injections.

## Ethical status and data

Being a review paper, no ethical oversight was required.

This data was presented as part of a panel discussion on injections at the SMA/ACSEP Conference in Melbourne in October 2024.

## Declaration of competing interest

The author has no direct financial conflicts, but declares an indirect conflict in that he is able to bill for injection prescription to patients (both PRP and corticosteroid) as part of his Sport & Exercise medicine practice.
